# Treatment of Strumous Disorders by Means of Preparation of Leaves of Walnut

**Published:** 1851-03

**Authors:** 

**Affiliations:** Physician at Angers


*Treatment of Strumous Disorders by means of Preparation of Leaves of Walnut.* By Dr. Negrier, Physician at Angers. — [ Dr. Negrier has published two memoirs, the first in 1841, and the second in 1844 on this subject. These consisted entirely of practical facts, without any theoretical view either of the disease or remedy, and the experience of five years, and that of several foreign physicians have given corroboration to the facts which the author then advanced. In this, the third memoir, he is desirous to avoid everything beyond a simple statement of facts, and the inferences which directly may be deduced from them. The following general conclusions are deduced by M. Negrier:]

Fifty-six patients, affected with scrofulous disease, in different forms, were treated by means of preparations of the walnut-tree leaves.

In this number, thirty one were cures, and did not afterwards disappoint expectation. Eighteen patients, without being completely cured, underwent a degree of amendment very remarkable in their condition; and the greatest number among them were in the course of being cured. Four patients have obtained no advantage from the use of the medicine, from the state of their sores. Among them was one who was remarkably strengthened, and who, at the time specified, seemed in the course of being cured, under the use of cod liver oil.

Four children died during treatment; but two were destroyed by tubercular phthisis, one by acute *encephalitis*, and the fourth by double pneumonia.

The treatment, by the preparations of walnut-tree leaves, has furnished to him results, which he regards as sufficiently advantageous, in order to justify their serious examination, and warrant their being subjected to the control of systematic observation, particularly by hospital physicians, who have better means than others of administering the remedy accurately and in comparison with other agents.

Experience has satisfied him that the long-continued use of walnut-tree leaves has never caused, in the economy, any unpleasant effects. This medicine, which may be placed in the class of slightly-aromatic bitters, possesses an efficacy nearly uniform in scrofulous disorders.

The first effect of the preparations of walnut-tree leaves is to augment the activity of digestion and circulation; to all the functions they impart a remarkable degree of energy. A natural question is, whether they exert any special action upon the lymphatic system? The facts recorded by the author lead him to think that they do. Under the influence of their use, the muscles become more firm, the skin acquires a ruddy tint, and speedily parts with its chlorotic paleness. It may be a subject of inquiry whether the walnut-tree leaf contains any other principle than tannin, which is, doubtless, not foreign to the tonic action of this vegetable.

As to comparison between this and other received methods of treatment, the condition in which most of the patients whose cases formed subjects of clinical observation were found, at the time of treatment, sufficiently prove, he maintains, that these different modes of management possessed no efficacy, when recourse was had to the preparation of walnut-tree leaves.

The preparations employed are the following:

The infusions of the walnut-tree leaves are made by placing a good amount (pinch) of these leaves, cut into two hundred and fifty grammes of boiling water. This infusion is sweetened with sugar, or with the syrup to be presently noticed. M. Negrier prescribed from two to three cupfuls of this infusion daily; and as many as five may be given.

The decoction of the leaves, which acts advantageously in the form of lotions, and as a topical application, in which the pledgets for dressing the sores are immersed, ought to be more strongly charged than the infusion. M. Negrier uses a small handful of leaves to a kilogramme of water, and boiling is continued from ten to fifteen minutes. This preparation is also very useful in the form of baths both local and general. Its efficacy is particularly remarkable when used by injection into fistulous passages. The extract is prepared with the walnut-tree leaves, by the method of displacement. By employing the dry leaves, the practitioner has in his power the means of renewing this preparation, as often as he requires, during all seasons; while, by using recent leaves, it is requisite to prepare it in too large quantity, and it is liable to undergo alteration. The syrup of the leaves of walnut-tree is prepared with the extract by mingling forty centigrammes with thirty grammes of simple syrup. By this means the practitioner knows what are the doses of the medicine which he employs. The syrup may also be prepared with the green leaves; and it is then more aromatic than that which is compounded with the extract; but it is impossible in this case to form a correct estimate of the quantity of the medicine which the patient daily takes. To young children he gives two or three teaspoonfuls of syrup in the course of twenty-four hours. The ordinary dose for adults is from thirty-two to forty grammes; and he has never gone beyond sixty-four grammes.

The pills of the extract of walnut-tree leaves are each twenty centigrammes of extract, rendered consistent by a sufficient amount of the powder of the leaves. Of these, two are taken daily; and he has never gone beyond four daily.

Lastly, in some cases, in which it may be useful to cause friction to be made upon the afflicted region, he employed an ointment composed of thirty grammes of extract of the leaves, forty grammes of lard, and fifteen centigrammes of essential oil of bergamot. The frictions require to be made gently, and for about fifteen minutes twice daily.

It is easy to understand, he observes in conclusion, that, considering the nature of the disease, the salutary effects of treatment are sometimes slow in appearing. He therefore recommends perseverance; and he expresses the opinion that, if in the hands of some practitioners the preparations of walnut-tree leaves have been administered without efficacy, the reason is, that either the patient or the physician became too soon tired with using them. It is, in short, requisite to persevere, when it is remembered, that, in order to obtain a durable cure, the physician has not only to oppose the effects of the malady, but also to effect a thorough change in the constitution of the individual.

[Having published his second memoir in 1844, M. Negrier completed the history of those patients given in the first paper, adding a list of persons treated by the remedy subsequent to the publication of the first memoir, and he gives the following inferences on the result of the observations of six years experience upon the mode of treatment:]—

1. Scrofulous disorders are in general radically cured by the use of preparations of walnut-tree leaves.

2. The action of this therapeutic agent possesses a sufficient degree of steadiness to enable the practitioner to reckon upon the cure of three-fourths of the patients treated by means of it.

3. The action of this mode of treatment is generally slow. It requires from twenty to fifty days according to the nature of the symptoms, and the constitution of the subjects, in order that the effects may be rendered sensible.

4. Patients cured by means of the preparations of walnut-tree leaves almost all retain the health which they have obtained under the influence of this treatment. Few relapses take place.

5. The effects produced by the internal use of the extract of walnut-tree leaves are at first general: the influence of this method of treatment is subsequently evinced upon the local symptoms.

6. In certain forms of scrofulous disorders, it is only after a long time that the efficacious action of this treatment is observed. This remark is particularly applicable to strumous glands not in a state of ulceration.

7. The preparations of walnut-tree leaves exert, on the contrary, a sufficiently speedy action upon the sores and the fistulous openings, whether they are maintained or not by caries of the bones, excepting in persons of dry and nervous temperament.

8. Till the present time, the instances of strumous opthalmia which M. Negrier has observed, have been certainly and more rapidly cured by this method of treatment than by any other.—*Edin. Med. and Surg. Journal, Oct.* 1. 1850. *p.* 271.

